# Supplementary material for: A p38α-BLIMP1 signalling pathway is essential for plasma cell differentiation
Source: Nat Commun. 2022 Nov 28;13:7321. doi: 10.1038/s41467-022-34969-0 (PMC9703440; doi:10.1038/s41467-022-34969-0)
Supplement: Supplementary file 1 — Supplementary Information [file 41467_2022_34969_MOESM1_ESM.pdf]

A p38α-BLIMP1 signalling pathway is essential for plasma cell differentiation

Jianfeng Wu[1,2,#], Kang Yang[1,#], Shaowei Cai[1,#], Xiaohan Zhang[1,#], Lichen Hu[1], Fanjia Lin[1], Su-qin Wu[2], Changchun Xiao[1], Wen-Hsien Liu[1]*, Jiahuai Han[1,2,3]*

[#]These authors contributed equally: Jianfeng Wu, Kang Yang, Shaowei Cai, Xiaohan Zhang.

*Correspondence authors.

Email: W.-H.L (whliu@xmu.edu.cn), J.H (jhan@xmu.edu.cn).

Supplementary figure legends

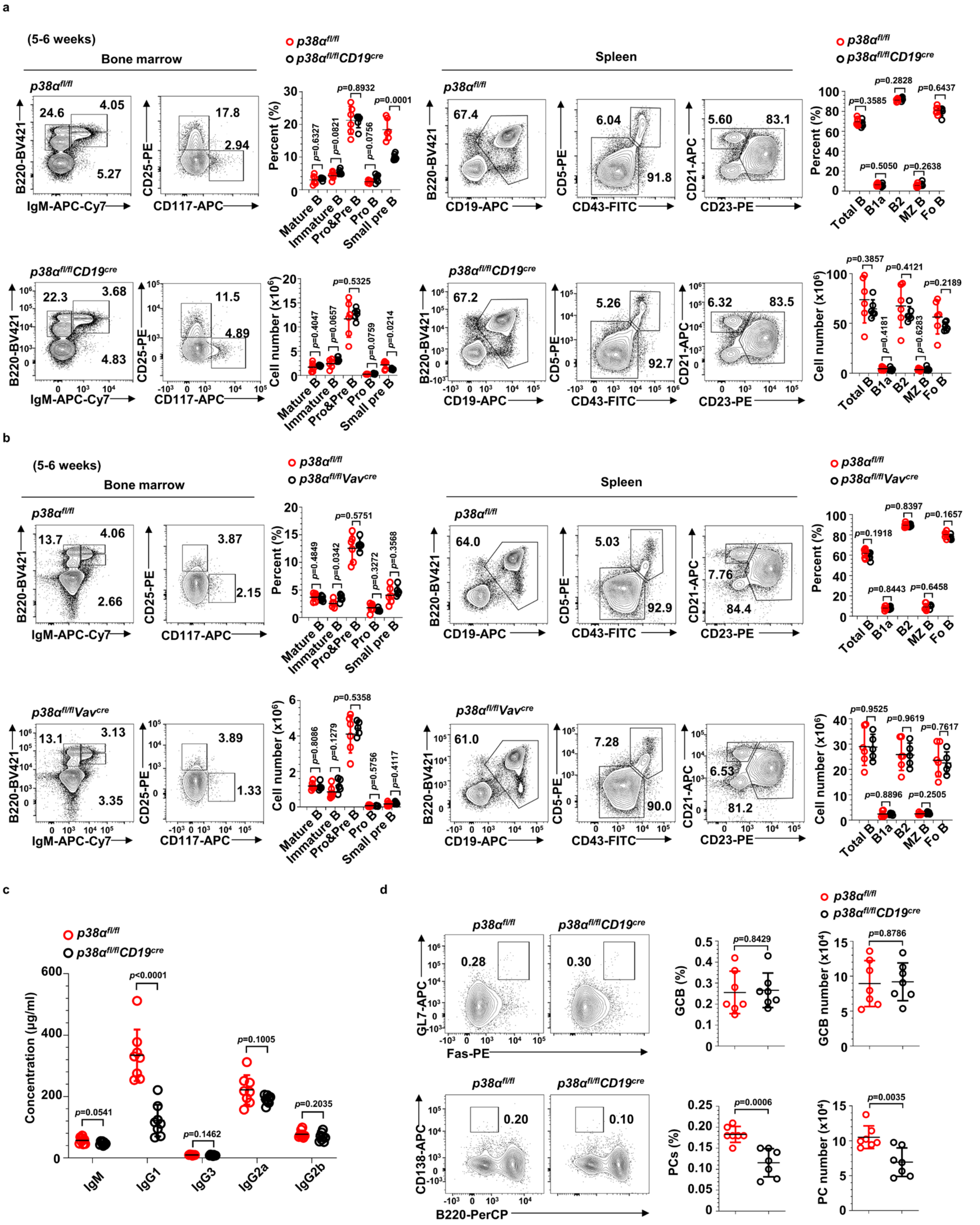

Supplementary Figure 1. The role of p38α in B cell development and antibody production.

(a) Flow cytometry analysis of indicated B cell subsets in the bone marrow and spleen of 5-6 weeks old *p38α^fl/fl CD19^cre* and *p38α^fl/fl* mice (n≥6 per group). Total B cells: $CD19^+$; B2 cells: $CD19^+CD5^{low}CD43^{low}$; B1a cells: $CD19^+CD5^{hi}CD43^{hi}$; MZ B cells:

$CD19^+CD21^{hi}CD23^{low}$; Fo B cells: $CD19^+CD21^{low}CD23^{hi}$; Mature B cells: $B220^{hi}IgM^+$; Immature B cells: $B220^{low}IgM^+$; Pro&Pre B cells: $B220^+IgM^-$; Pro B cells: $B220^+IgM^-CD25^-CD117^+$; Small pre B cells: $B220^+IgM^-CD25^+CD117^-$. **(b)** Flow cytometry of indicated B cell subsets as described in (a) in the bone marrow and spleen of 5-6 weeks old $p38\alpha^{fl/fl}Vav^{cre}$ and $p38\alpha^{fl/fl}$ mice (n≥5 per group). **(c)** Serum immunoglobulin (Ig) levels in $p38\alpha^{fl/fl}CD19^{cre}$ and $p38\alpha^{fl/fl}$ mice (8-10 weeks, n=8 per group) were determined by ELISA. **(d)** Flow cytometry analysis of GCB cells and PCs in the spleen of non-immunized $p38\alpha^{fl/fl}CD19^{cre}$ and $p38\alpha^{fl/fl}$ mice (8-10 weeks, n=7 per group). Summary of the percentage and number of GCB cells and PCs. Each symbol represents an individual mouse. Small horizontal lines indicate the mean (± s.d.). Data were analyzed by two-tailed unpaired t-tests. Source data are provided as a Source Data file.

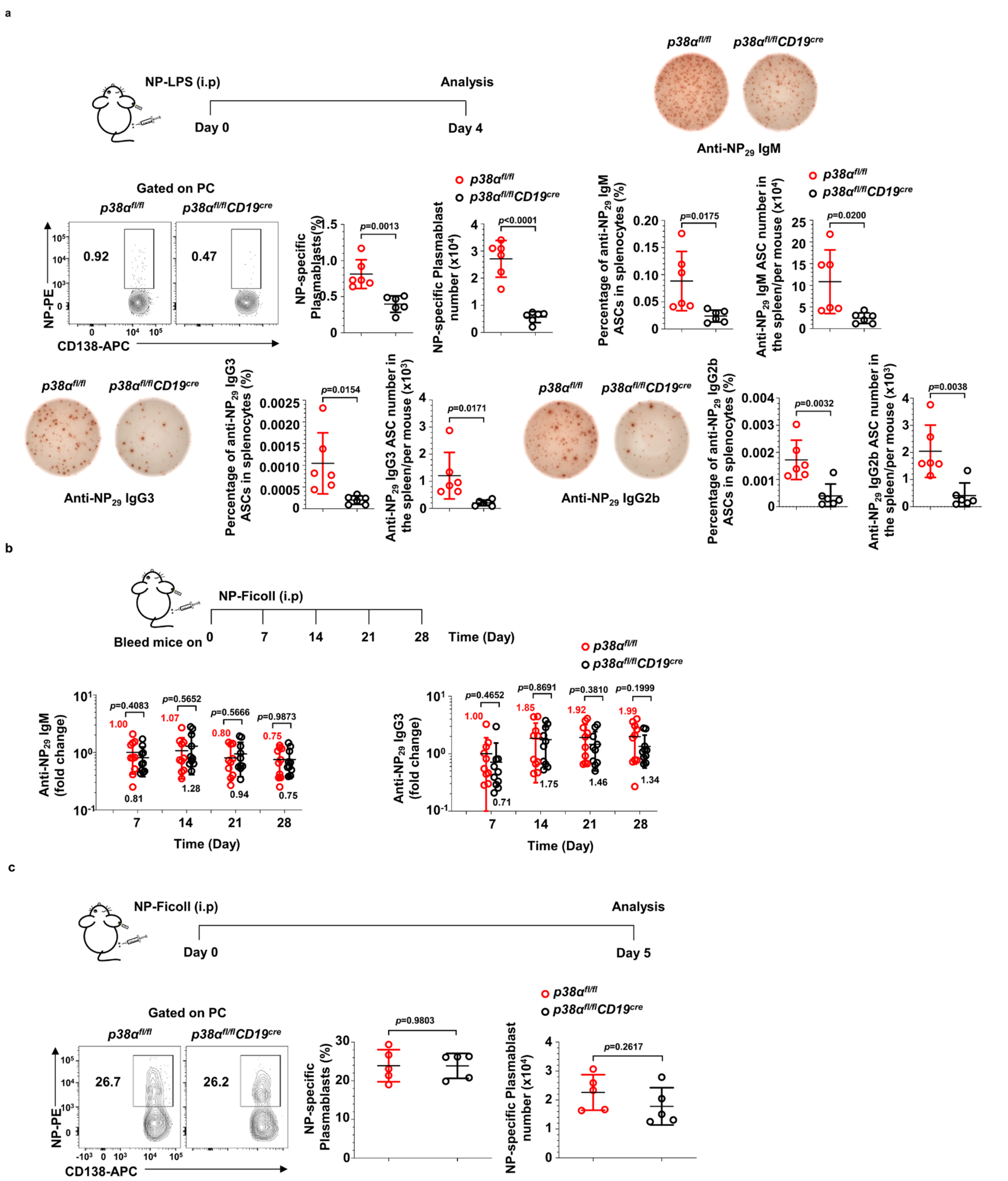

Supplementary Figure 2. The role of p38α in T cell-independent antibody responses.

(a) Flow cytometry analysis of NP-specific PBs (B220^{low}CD138^{+}NP^{+}) in the spleen of *p38α$^{fl/fl}$CD19^{cre}* and *p38α$^{fl/fl}$* mice (8-10 weeks, n=6 per group) at day 4 post immunization with NP-LPS (i.p). NP-specific antibody secreting cells (ASCs) were measured by ELISpot assay. **(b)** ELISA analysis of serum NP-specific antibody concentration in *p38α$^{fl/fl}$CD19^{cre}* and *p38α$^{fl/fl}$* mice immunized with NP-Ficoll (i.p) at indicated time points (n=10 per group). **(c)** Flow cytometry analysis of NP-specific PBs

($B220^{low}CD138^{+}NP^{+}$) in the spleen at day 5 post immunization with NP-Ficoll (i.p) (n=5 per group). Each symbol represents an individual mouse. Small horizontal lines indicate the mean (± s.d.). Data were analyzed by two-tailed unpaired t-tests. Source data are provided as a Source Data file.

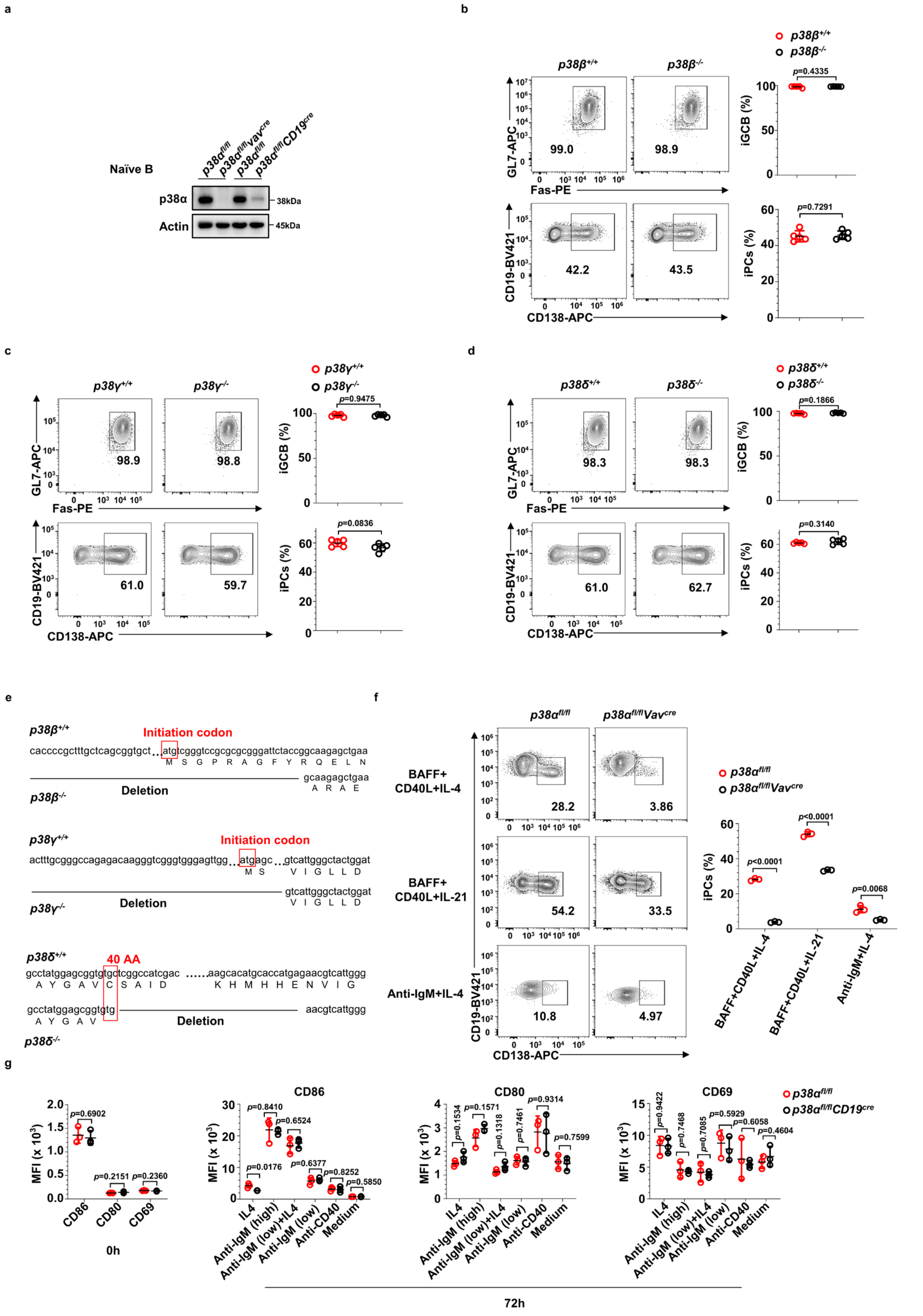

Supplementary Figure 3. The role of p38 MAPK family members in iPCs generation.

(a) Immunoblot analysis of p38α and Actin in naïve B cells of indicated genotypes. **(b to d)** Splenic naïve B cells from *p38β*$^{-/-}$, *p38γ*$^{-/-}$ or *p38δ*$^{-/-}$ and WT mice were cultured

in iPC differentiation system. iPCs ($CD19^+CD138^+$) were analyzed by flow cytometry at day 8 (n=5 per group). **(e)** Portions of the CDS sequences of *p38β*, *p38γ* and *p38δ* of the $p38\beta^{-/-}$, $p38\gamma^{-/-}$ and $p38\delta^{-/-}$ mice used in this study. **(f)** *p38α* KO and WT splenic naïve B cells were stimulated with BAFF+CD40L+IL-4 (8 days), BAFF+CD40L+IL-21 (6 days), and anti-IgM+IL-4 (4 days), and iPCs ($CD19^+CD138^+$) were analyzed by flow cytometry (n=3 per group). **(g)** Splenic naïve B cells from $p38\alpha^{fl/fl}CD19^{cre}$ and $p38\alpha^{fl/fl}$ mice were stimulated with IL-4 (5 ng/ml), anti-IgM (high concentration, 10 μg/ml), anti-IgM (low concentration, 1 μg/ml) + IL-4 (5 ng/ml), anti-IgM (low concentration, 1 μg/ml), anti-CD40 (5 μg/ml) for 3 days. MFI of activation markers (CD86, CD80 and CD69) was analyzed by flow cytometry (n=3 per group). Medium was used as a negative control. Data were representative of at least three independent experiments. Each symbol represents a representative sample. Small horizontal lines indicate the mean (± s.d.). Data were analyzed by two-tailed unpaired t-tests. Source data are provided as a Source Data file.

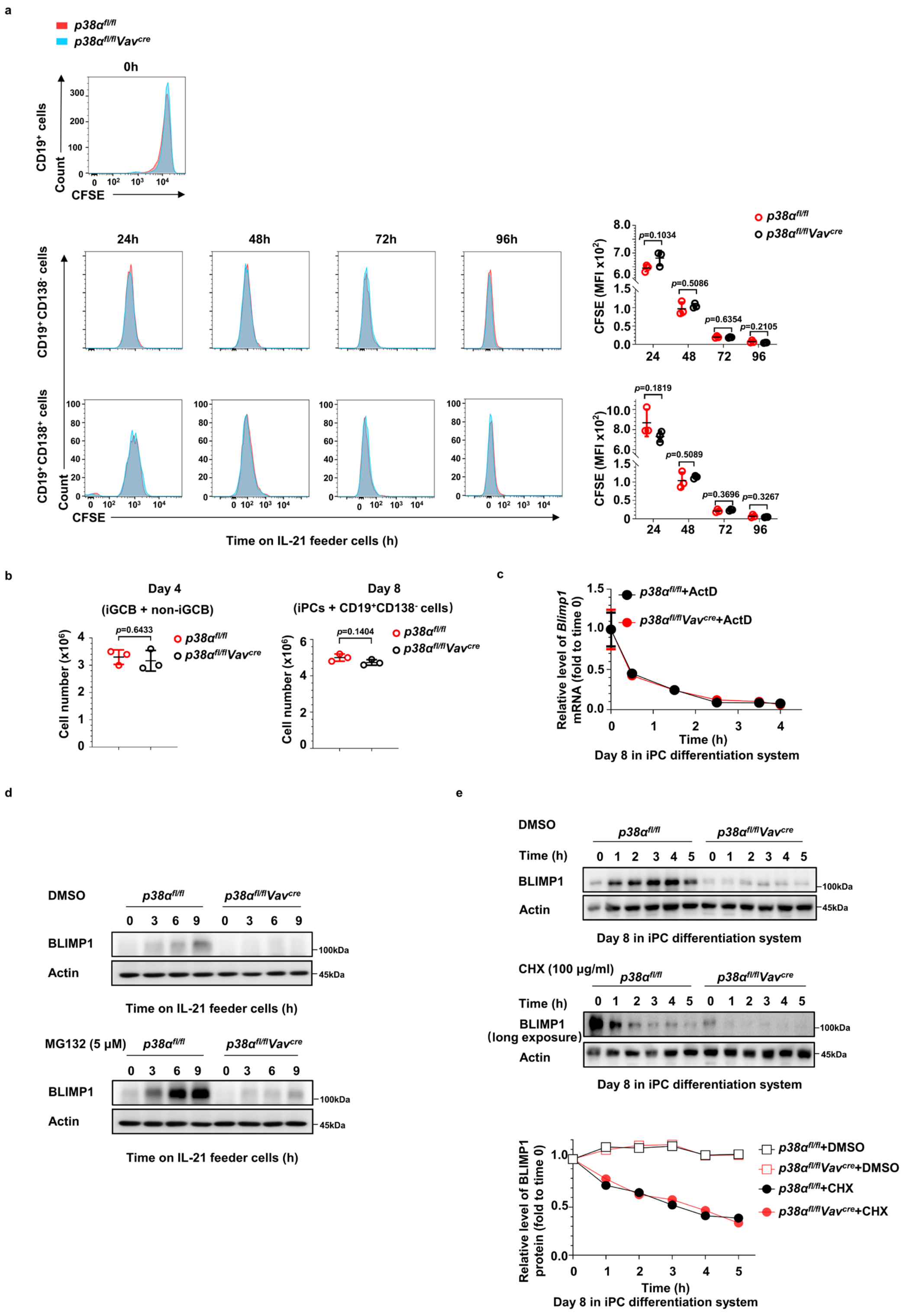

Supplementary Figure 4. p38α regulates BLIMP1 expression, but not B cell proliferation.

(a) CFSE-labeled *p38α* KO and WT iGCB cells were transferred onto IL-21 feeder cells. CFSE histogram and MFI of CFSE in iPCs (CD19^{+}CD138^{+}) and CD138^{-} B cells

($CD19^+CD138^-$) were analyzed at indicated time points (n=3 per group). **(b)** Total numbers of B cells ($CD19^+$) at day 4 and 8 in the iPC differentiation system (n=3 per group). **(c)** *p38α* KO and WT B cells cultured in the iPC differentiation system for 8 days were treated with actinomycin D (50 μM). *Blimp1* mRNA levels were measured by quantitative RT-PCR at indicated time points (n=3 per group). **(d)** Immunoblot analysis of BLIMP1 and Actin expression in cultured *p38α* KO and WT iGCB cells after being transferred onto IL-21 feeder cells and treated with MG132 (5 μM) or DMSO for indicated periods of time. **(e)** Immunoblot analysis of BLIMP1 and Actin expression in *p38α* KO and WT B cells cultured in the iPC differentiation system and treated with cycloheximide (CHX, 100 μg/ml) or DMSO for indicated periods of time at day 8 of culture, and the densities of the protein bands was semi-quantitated. Data were representative of at least three independent experiments. Each symbol represents a representative sample. Small horizontal lines indicate the mean (± s.d.). Data were analyzed by two-tailed unpaired t-tests. Source data are provided as a Source Data file.

a

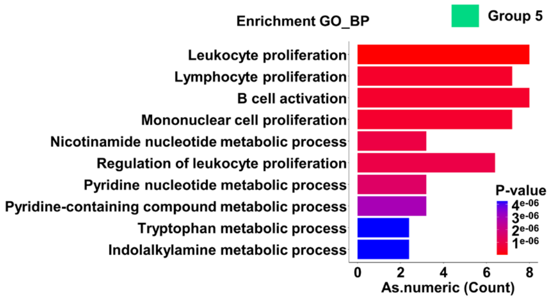

Supplementary Figure 5. GO enrichment analysis of gene sets from Group 5.

(a) GO enrichment analysis result of gene sets from Group 5 in Fig. 4c (listed in Supplementary Table. 1). Data were analyzed by one-side hypergeometric test.

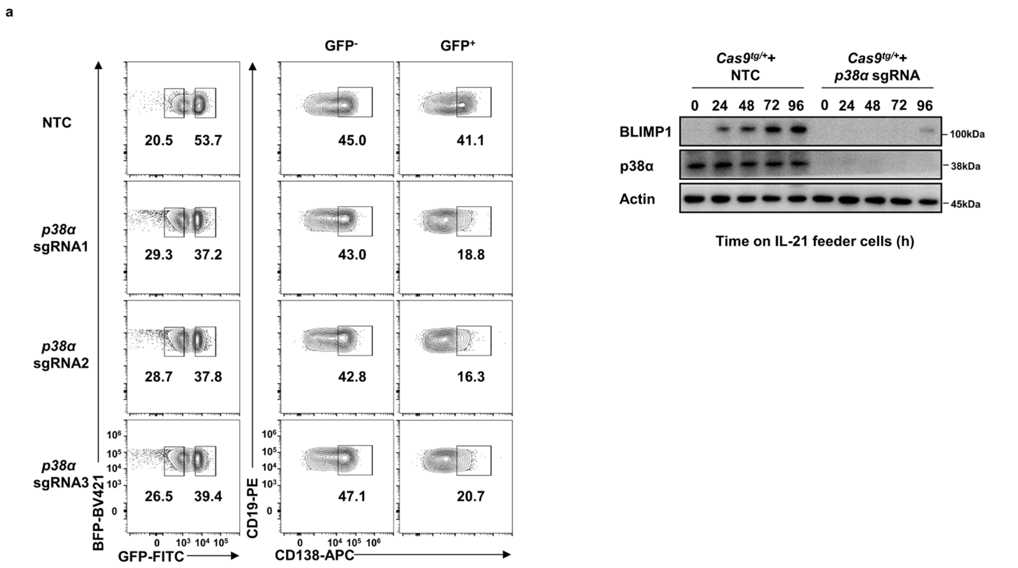

Supplementary Figure 6. High efficiency of the CRISPR/Cas9 screen system.

(a) Cultured B cells from Cas9$^{tg/+}$ mice (Cas9-IRES-GFP transgenic mice, GFP^{+}) and WT mice (GFP^{-}) were transduced with retroviruses encoding sgRNAs targeting *p38α* at day 2 of iPC culture. Percentage of iPCs in retrovirus-transduced Cas9-expressing B cells (CD19^{+}GFP^{+}BFP^{+}CD138^{+}) was analyzed by flow cytometry at day 8. Immunoblot analysis of BLIMP1, p38α and Actin expression in *p38α* or NTC sgRNA expressing B cells after being transferred onto IL-21 feeder cells of indicated time. Data were representative of at least three independent experiments. Source data are provided as a Source Data file.

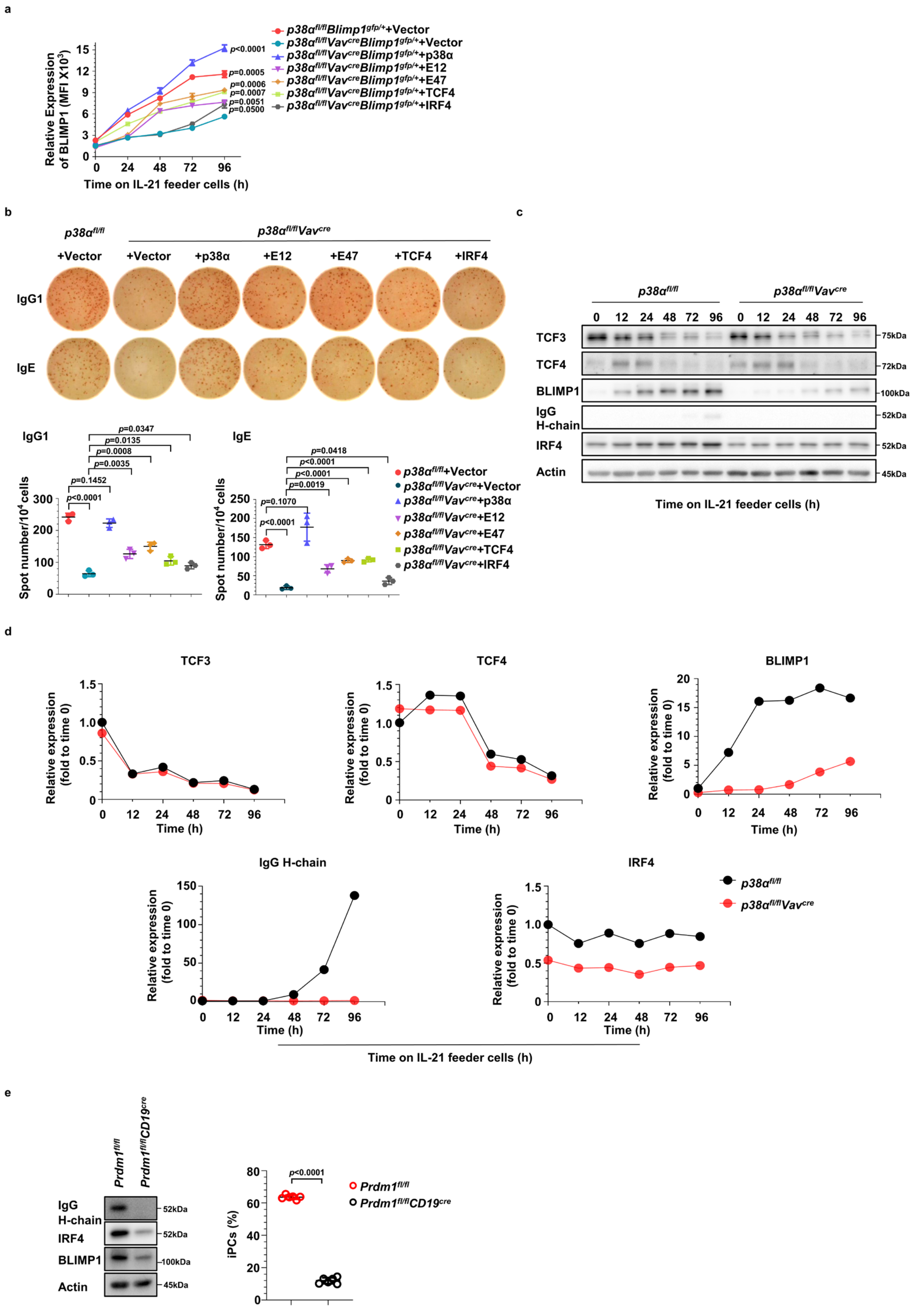

Supplementary Figure 7. p38α regulates BLIMP1 expression and ASCs generation through regulating activation of TCF3, TCF4 and IRF4.

(a) TCF3 (E12 or E27), TCF4, and IRF4 were ectopically expressed in cultured *p38α*$^{fl/fl}$*Vav*^{cre}*Blimp1*$^{gfp/+}$ B cells via retroviral transduction (BFP^{+}). MFI of GFP

(indicating the expression level of BLIMP1) in retrovirus-transduced B cells ($CD19^+$ BFP^+) was measured by flow cytometry at indicated time points (n=3 per group). **(b)** Cultured *p38α* KO and WT B cells were transduced with retroviruses encoding TCF3 (E12 or E47), TCF4, or IRF4 at day 2. IgG1 and IgE antibody secreting cells (ASCs) in retrovirally transduced B cells ($CD19^+GFP^+$) were sorted and analyzed by ELISpot assay at day 8 (n=3 per group). **(c and d)** Immunoblot analysis of TCF3, TCF4, IRF4, BLIMP1, IgG H-chain and Actin in *p38α* KO and WT iGCB cells transferred onto IL-21 feeder cells at indicated time points, and the densities of the protein bands was semi-quantitated. The western blot samples were derived from the same experiment and that gels/blots were processed in parallel. **(e)** Immunoblot analysis of IgG H-chain, IRF4, BLIMP1, and Actin in *Blimp1* KO and WT cells at day 8 in iPC culture. Flow cytometry analysis of the percentage of iPCs ($CD19^+CD138^+$) at day 8 of culture (n=6 per group). Data were representative of at least three independent experiments. Each symbol represents a representative sample. Small horizontal lines indicate the mean (± s.d.). Data were analyzed by two-tailed unpaired t-tests. Source data are provided as a Source Data file.

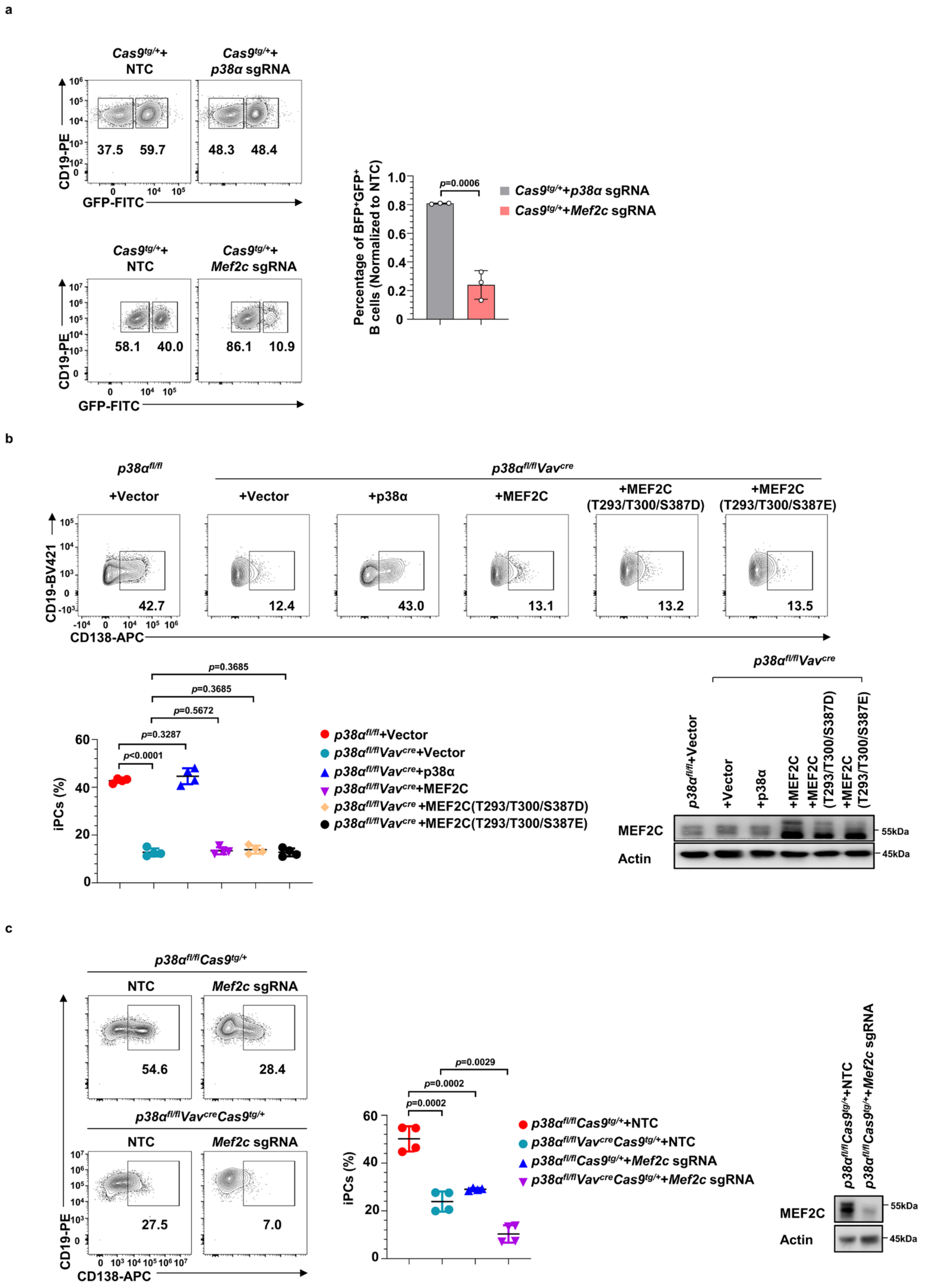

Supplementary Figure 8. MEF2C was not downstream of p38α in iPC differentiation.

(a) Retroviruses encoding NTC sgRNA or sgRNA targeting to *p38α* or *Mef2c* (BFP^{+}) were transduced into cultured B cells (Cas9-expressing B cells (GFP^{+}) mixed with WT B cells (GFP^{-}) at 1:1 ratio) at day 2 in iPC differentiation system. The percentages of cultured $BFP^{+}GFP^{-}$ B cells and $BFP^{+}GFP^{+}$ B cells of indicated genotypes were analyzed

at day 8 by flow cytometry, and the ratio of *p38α* or *Mef2c* sgRNA transduced BFP^+GFP^+ B cells to NTC transduced BFP^+GFP^+ B cells was shown (n=3 per group). NTC, non-targeting control. **(b)** Cultured *p38α*$^{-/-}$ B cells were transduced with retroviruses encoding MEF2C or related mutants at day 2. iPCs among retrovirus-transduced B cells ($CD19^+GFP^+CD138^+$) were analyzed by flow cytometry, and MEF2C and Actin in retrovirus-transduced B cells ($CD19^+GFP^+$) were analyzed by immunoblot at day 8 in iPC culture (n=4 per group). **(c)** $p38\alpha^{fl/fl}Vav^{cre}Cas9^{tg/+}$ and $p38\alpha^{fl/fl}Cas9^{tg/+}$ iGCB cells were transferred onto IL-21 feeder cells and transduced with retroviruses encoding sgRNA targeting *Mef2c* 1 day later. The percentage of iPCs in retrovirus-transduced B cells ($CD19^+GFP^+BFP^+CD138^+$) was analyzed by flow cytometry, and MEF2C and Actin in retrovirus-transduced B cells were analyzed by immunoblot 3 days after transduction (n=4 per group). NTC, non-targeting control. Data were representative of at least three independent experiments. Each symbol represents a representative sample. Small horizontal lines indicate the mean (± s.d.). Data were analyzed by two-tailed unpaired t-tests. Source data are provided as a Source Data file.

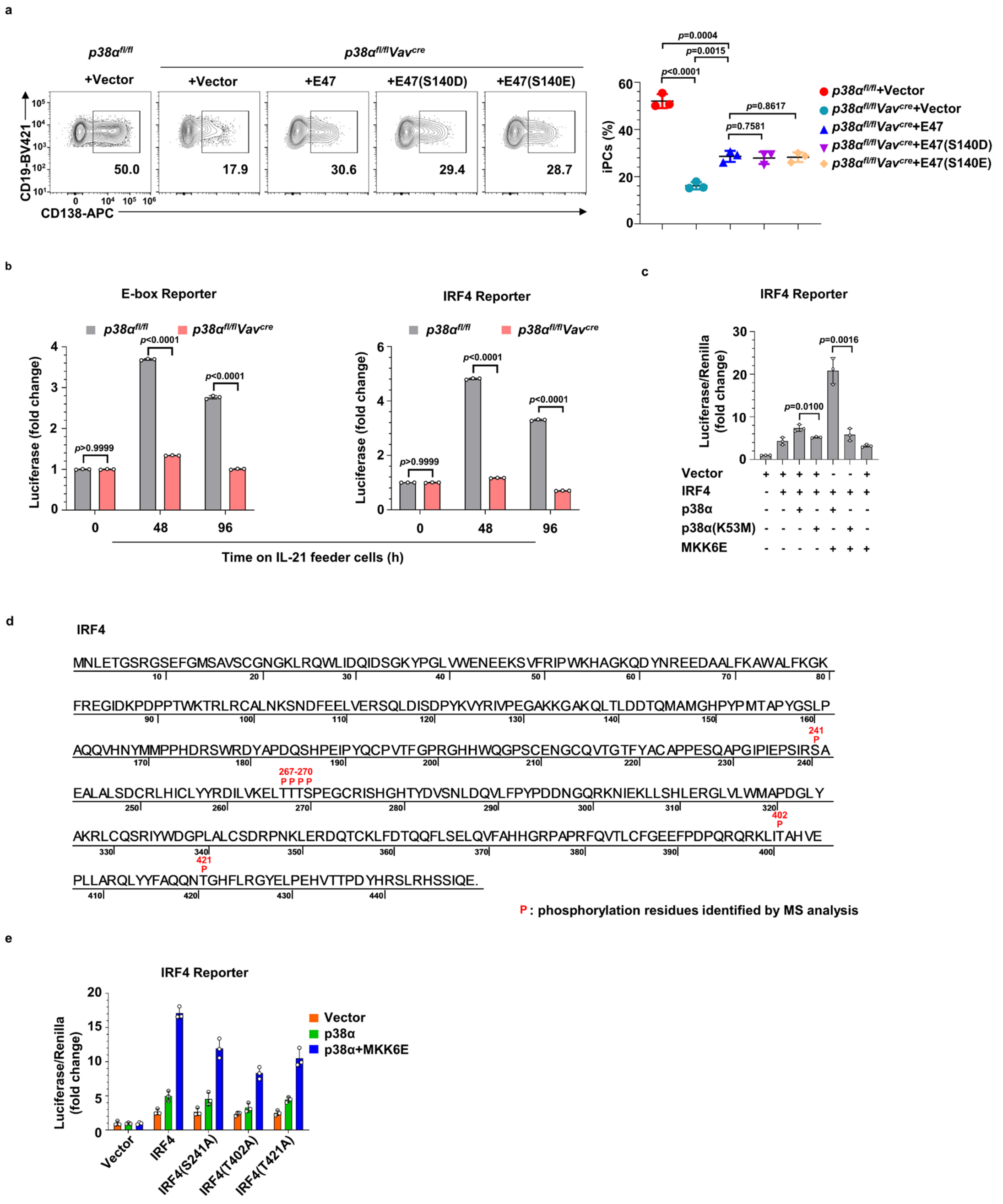

Supplementary Figure 9. The activation of TCF3, TCF4 and IRF4 downstream of p38α in PC differentiation.

(a) Cultured *p38α* KO and WT B cells were transduced with retroviruses encoding E47 S140D or S140E at day 2 in iPC culture. The percentage of iPCs in retrovirus-transduced B cells ($CD19^{+}GFP^{+}CD138^{+}$) was analyzed by flow cytometry at day 8 (n=3 per group). **(b)** Cultured *p38α* KO and WT B cells were transduced with indicated luciferase reporter at day 2 in iPC culture, and the luciferase activity in cultured B cells was analyzed at indicated time (n=3 per group). **(c)** *p38*$^{-/-}$ 293A cells were transfected

with indicated plasmids, and the luciferase activity of IRF4 was examined at 24 hours after transfection (n=3 per group). **(d)** Schematic outline of the p38α phosphorylated residues on IRF4 identified by MS (red). **(e)** *p38*$^{-/-}$ 293A cells were transfected with indicated plasmids and the luciferase activity of IRF4 or related mutants was examined at 24 hours after transfection (n=3 per group). Data were representative of at least three independent experiments. Each symbol represents a representative sample. Small horizontal lines indicate the mean (± s.d.). Data were analyzed by two-tailed unpaired t-tests. Source data are provided as a Source Data file.

a

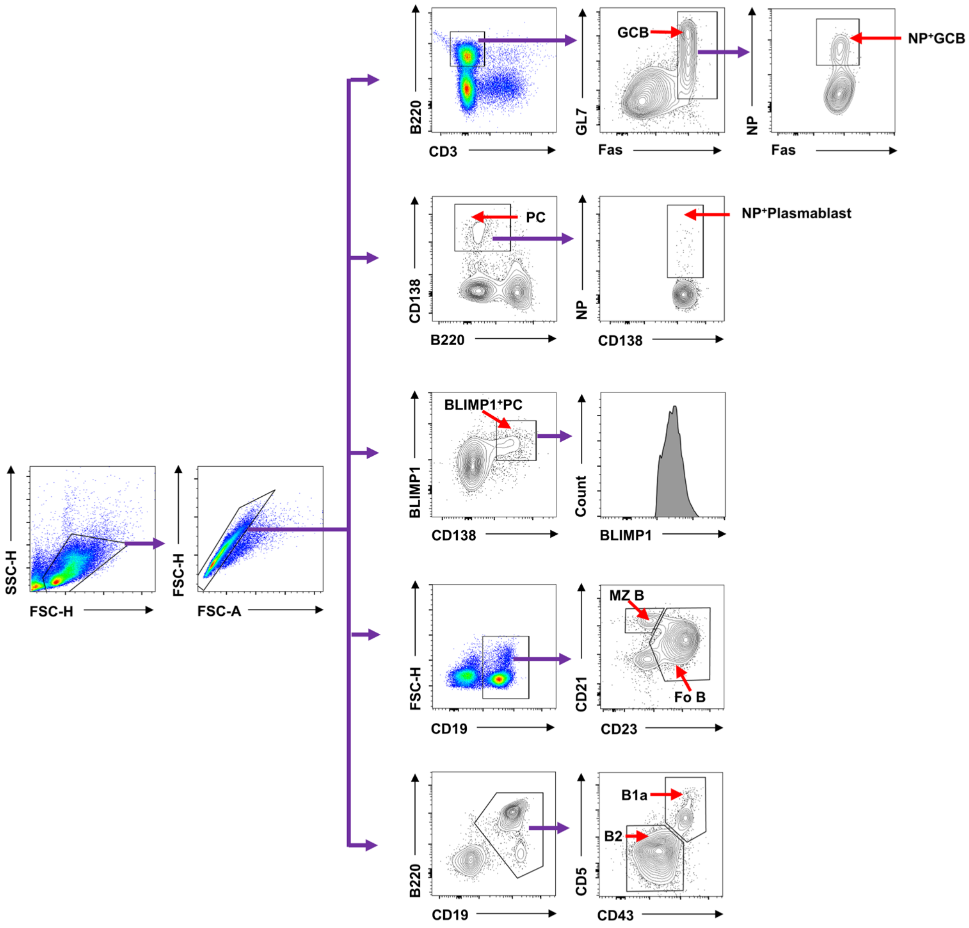
SSC-H
FSC-H
FSC-H
FSC-A
B220
CD3
GL7
Fas
GCB
NP
Fas
NP⁺GCB
CD138
B220
PC
NP
CD138
NP⁺Plasmablast
BLIMP1
CD138
BLIMP1⁺PC
Count
BLIMP1
FSC-H
CD19
CD21
CD23
MZ B
Fo B
B220
CD19
CD5
CD43
B1a
B2

b

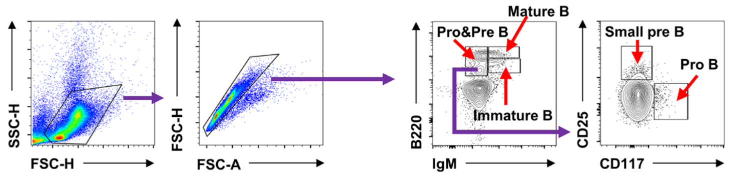
SSC-H
FSC-H
FSC-H
FSC-A
B220
IgM
Mature B
Pro&Pre B
Immature B
CD25
CD117
Small pre B
Pro B

c

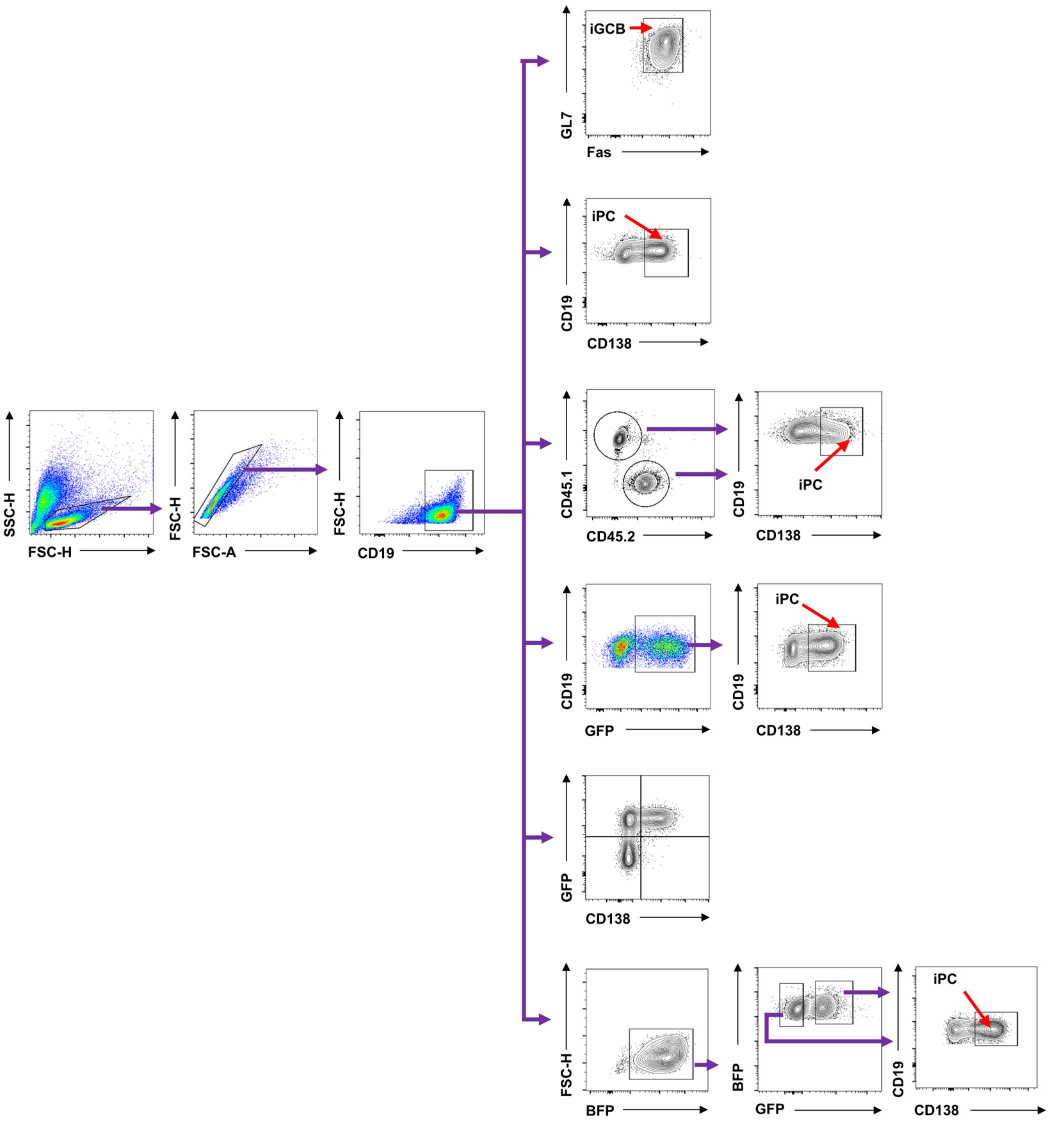

Supplementary Fig 10. FACS gating strategies.

(a) FACS gating strategies for splenic B cells *in vivo*, related to Fig. 1a, 1c, 3d; Supplementary Fig. 1a, 1b, 1d, 2a, 2c. **(b)** FACS gating strategies for bone marrow B cells *in vivo*, related to Supplementary Fig. 1a, 1b. **(c)** FACS gating strategies for splenic B cells *in vitro*, related to Fig. 2a, 2c, 2e, 3a, 3b, 4a, 5c, 5e-5g, 6a, 6c, 6d, 6f, 6g, 7c, 8a-8c, 9a-9d; Supplementary Fig. 3b-3d, 3f, 6a, 7e, 8a-8c, 9a.

Supplementary Table 1. Genes in classified 8 groups of Fig. 4C.

Group	Group Description	Genes name
Group1	Expression of genes decreased in *p38α* KO iPCs, restored by p38α or BLIMP1 overexpression.	1700012L04Rik/1810011O10Rik/1810041H14Rik/2500002B13Rik/A430035B10Rik/Ankrd55/Anxa8/Apoops/Arpp21/Arrdc3/Atat1/B3gnt9/Bach1/Bcl2l1/Brwd1/Btg2/Btg3/Cacna1e/Cacna1h/Calcoco1/Ccdc17/Ccpg1/Cd28/Cd55/Cd93/Chac1/Chid1/Chst15/Coro2b/Cox4i2/Creb3l2/Creg1/Creld2/Ctsd/Ctsl/Cysltr2/D17H6S56E5/Defa6/Derl3/Dusp1/Dusp5/Dusp9/Eaf2/Egr1/Egr3/Epcam/Evi5/Fabp2/Fads3/Farp2/Fgfbp3/Firre/Fkbp11/Fndc3b/Fos/Fosb/Foxp1/Gm12504/Gm4013/Gm4070/Gm5547/Gpm6a/H1/H2T24/Hepacam2/Hist1h2al/Hist2h4/Hist3h2a/Hmces/Hsd11b1/Icam2/Ifnar2/Il5ra/Il6st/Irs2/Isg15/Isg20/Itm2b/Jchain/Kcnk6/Kctd12/Kdelr3/Kdm5b/Laptm4a/Lgmn/Ly6c2/Malat1/Mettl21b/Mfsd4a/Mir22hg/Myo1h/Mzb1/Neat1/Nfkbiz/Nr1d2/Ntng2/Nucb2/Oosp1/Os9/Osgin1/Pdia4/Pkd2l2/Pld3/Plxnb2/Pon3/Prap1/Prdx4/Prg2/Rabac1/Raph1/Rnaset2a/Rsl1/S100a6/Scarna6/Scgb1a1Sdc1/Selm/Sema4g/Sepp1/Serpina3f/Serpini1/Siae/Ski/Slpi/Snord22/Spint2/Ssr4/Tcp11l2/Thbd/Thbs1/Tmcc3/Tmem170/Tmem176a/Tmem176b/Tnfrsf17/Tnnt3/Tns3/Trp53inp1/Txndc5/Wipi1/Xbp1/Zbp1/Zdhhc14/Zfp36/Zfyve28
Group2	Expression of genes decreased in *p38α* KO iPCs, restored by p38α but not BLIMP1 overexpression.	0610039H22Rik/1810026B05Rik/2310001H17Rik/2310045n01rik/mef2b/A930006K02Rik/Acta1/Ada/Alpl/Arfgef3/Bhlha15/C1galt1/Capn5/Cd274/Cecr2/Chst7/Cpeb3/Ctla4/Ctse/Defa6/Dennd5b/Dnajb9/E130215H24Rik/Edem2/Edem3/Emilin1/Entpd1/F2rl1/Fads2/Fam129c/Filip1l/Fkbp2/Fndc3aFut8/Gca/Gm11423Gm5535/Gpr160/H1f0/Hid1/Hist1h1cHsp90b1/Hspa5/Ift20/Lipg/Lman1/Lncpint/Ly6k/Mapk14/Mcts2/Nuggc/Pafah1/Peli1/Pipox/Prap1/Reln/Rgcc/S100a1/Scarna6/Scgb1a1/Sec11c/Smad3/Snord22/Tmem52/Ttc41/Txndc11/Vsir
Group3	Expression of genes decreased in *p38α* KO iPCs, restored by BLIMP1 but not p38α overexpression.	1810011O10Rik/Ccl3/Gbp2b/Gzma/Igfbp6/S100a6/Sparc/Tgtp1/Thbs1
Group4	Expression of genes decreased in *p38α* KO iPCs, restored by neither p38α nor BLIMP1 overexpression.	A430035B10Rik/A930006K02Rik/Acta1/Cd274/Chst7/D330023K18Rik/Defa6/Eif3j2/Fabp2/Gbp6/Glcci1/Gm11423/Gm4013/Gm5547/Hist1h2ah/Hist1h2al/Hist1h4f/Hist2h2ac/Hist2h4/Igtp/Irgm2/Isg15/Ly6k/Plac8/Prap1/Prelid2/Rnaset2a/Rps15a-ps6/Scgb1a1/Serpina3f/Snord22/Tgtp2/Tmem52
	Expression of genes increased in *p38α*	1110032F04Rik/2900026A02Rik/Ackr2/Adgrg5/Ahr/Aicda/Arhgap26/Asb2/B3gnt5/B4galnt4/B4galt5/Bank1/Bcl11a/Bfsp2/B

Group5	KO iPCs, restored by p38α or BLIMP1 overexpression.	lk/Btbd6/Cd22/Cd86/Cdca7/Ceacam1/Cetn4/Chst10/Clec2f/Cotl1/Dkk3Dok2/Evl/Ffar2/Flt3/Gatm/Gatsl3/Gimap7/Gnb5/Gpr146/Haao/Hhex/Hs3st1/Ido2/Ifitm1/Il1r2/Iqgap2/Itpr1/Ksr1/Kynu/Lmo2/Lrrc32/Lrrk2/Lta/Ly86/Mbp/Mpp2/Mt3/Myo1f/Nbeal2/Ncf1/Neurl2/Nkain1/Pax5/Phactr4/Phf11a/Phlpp1/Pla2g7/Rbm38/Rnf183/Rps19-ps3/Sbk1/Snhg10Snora64/Snord15b/Sorl1/Spib/Susd3/Tespa1/Tlr9/Tox/Trabd2b/Usp25/Zbtb32
Group6	Expression of genes increased in *p38α* KO iPCs, restored by p38α but not BLIMP1 overexpression.	1810043G02Rik/Ackr3/Aff3/Aim1/Arl10/B630019K06Rik/Batf3/Bcl2/Bcl2l12/Bin1/Brd3/Camk2b/Capn2/Cbx8/Ccr7/Cd300lf/Cdk6/Cfp/Ciita/Clstn1/Cmpk2/Cnn2/Cpne2/Cxx1a/Cxx1b/Cyb561a3/Dpp4Echdc3/Eid1/Ell3/Fam167a/Fam213a/Fam234b/Fam57a/Fcer2a/Flna/Fscn1/Fut4/Gm5861/Gm6455/Gnb4/Gypc/H2-M5/H2-Oa/Hadh/Htr7/I730030J21Rik/Ifi203/Il18r1/Il18rap/Il27Il9r/Irf2bpl/Itgb7/Jag2/Jdp2Jmjd7/Kctd17/Kif5c/L3mbtl3/Lacc1/LbhLrmp/Map2k6/Mfhas1/Msc/Msrb3/Mx1/Myo1e/Nckipsd/Ndufaf6/Nek6/Nrp2/Ntpcr/Olfr60/P2ry13/Palm/Peg13/Phc2/Phf11b/Pik3ap1/Plscr1/Prkag2/Ptpn22/Rcsd1/Rnf157/S100a11/Siglecg/Specc1/Spire1/Srpk3/Syt11/Tesc/Tlr12/Tm6sf1/Tmem229b/Tmem243/Tnfrsf8/Tnfsf4/Tnp2/Tpm3/Traf1/Trib2/Trp73/Tspan33/Tubb2b/Tubb3/Twf2/Vim/Zbtb18/Zbtb20/Zmiz1
Group7	Expression of genes increased in *p38α* KO iPCs, restored by BLIMP1 but not p38α overexpression.	Mnd1/Snord17
Group8	Expression of genes increased in *p38α* KO iPCs, restored neither p38α nor BLIMP1 overexpression.	5830417I10Rik/Dnajc12/Eif3j1/Itm2a/Mfsd13a/Pet117/Psd3/Sfxn2/Snord15a/Trdmt1
