# Supplementary material for: A p38α-BLIMP1 signalling pathway is essential for plasma cell differentiation
Source: Nat Commun. 2022 Nov 28;13:7321. doi: 10.1038/s41467-022-34969-0 (PMC9703440; doi:10.1038/s41467-022-34969-0)
Supplement: Supplementary file 3 — Description of Additional Supplementary Files [file 41467_2022_34969_MOESM3_ESM.pdf]

Description of Additional Supplementary Files

File Name: Supplementary Data 1
Description: Different expressed genes in p38α WT and KO iGCB cells and iPCs (Fig. 2B)

File Name: Supplementary Data 2
Description: sgRNA sequences target to p38α and its substrates

File Name: Supplementary Data 3
Description: TCF3(E47), TCF4, IRF4 phosphorylation sites by p38α identified by MS
