# Supplementary material for: A p38α-BLIMP1 signalling pathway is essential for plasma cell differentiation
Source: Nat Commun. 2022 Nov 28;13:7321. doi: 10.1038/s41467-022-34969-0 (PMC9703440; doi:10.1038/s41467-022-34969-0)
Supplement: Supplementary file 7 — Reporting Summary [file 41467_2022_34969_MOESM7_ESM.pdf]

nature portfolio

Corresponding author(s): Jiahuai Han
Last updated by author(s): Oct 25, 2022

Reporting Summary

Nature Portfolio wishes to improve the reproducibility of the work that we publish. This form provides structure for consistency and transparency in reporting. For further information on Nature Portfolio policies, see our Editorial Policies and the Editorial Policy Checklist.

Statistics

For all statistical analyses, confirm that the following items are present in the figure legend, table legend, main text, or Methods section.

n/a	Confirmed	
☐	☒	The exact sample size (*n*) for each experimental group/condition, given as a discrete number and unit of measurement
☐	☒	A statement on whether measurements were taken from distinct samples or whether the same sample was measured repeatedly
☐	☒	The statistical test(s) used AND whether they are one- or two-sided<br>*Only common tests should be described solely by name; describe more complex techniques in the Methods section.*
☒	☐	A description of all covariates tested
☐	☒	A description of any assumptions or corrections, such as tests of normality and adjustment for multiple comparisons
☐	☒	A full description of the statistical parameters including central tendency (e.g. means) or other basic estimates (e.g. regression coefficient) AND variation (e.g. standard deviation) or associated estimates of uncertainty (e.g. confidence intervals)
☐	☒	For null hypothesis testing, the test statistic (e.g. *F*, *t*, *r*) with confidence intervals, effect sizes, degrees of freedom and *P* value noted<br>*Give P values as exact values whenever suitable.*
☒	☐	For Bayesian analysis, information on the choice of priors and Markov chain Monte Carlo settings
☒	☐	For hierarchical and complex designs, identification of the appropriate level for tests and full reporting of outcomes
☒	☐	Estimates of effect sizes (e.g. Cohen's *d*, Pearson's *r*), indicating how they were calculated

Our web collection on statistics for biologists contains articles on many of the points above.

Software and code

Policy information about availability of computer code

Data collection

1. Mass spectrometry was performed with Orbitrap Fusion Lumos (Thermo) coupled with an ultra-high pressure nano-flow chromatography system (U3000, Thermo).
2. RNA-seq: Illumina Hi-seq system was used for sequencing of the library.
3. Flow cytometry was performed with BD fortesa X20 (analysis) and BD Aril-FUSION (sorting);
4. Electron microscopy was performed with Hitachi, HT-7800;
5. RT-RCR was performed with Bio-Rad CFX96tm Real-time system.
6. Microscopic images were collected with Leica TCS SP8 DLS;

Data analysis

1.Mass spectrometry were analyzed using Proteome Discovery software (version: 2.2.0.388);
2.RNA-seq: Raw data (raw reads) of Fastq format were processed by Fastp software (version: 0.19.7) to remove low-quality reads. Subsequent analyses were based on high quality clean data. Reads were aligned to the reference genome using STAR (version: 020201). Cufflinks (version: 2.2.1) was used to count the read numbers mapped to each gene.
3. Flow cytometry data were analysed using FlowJo software (version: 10.6.2);
4. RT-PCR data were analyzed using Bio-Rad CFX Manager software (version: 1.0.1035.131).
5. Statistical analyses were performed using GraphPad Prism (version: 8.0.2) and SPSS (version: 27.0).
6. Intensities of blots were quantified with Image J software (version: 1.8.0).

For manuscripts utilizing custom algorithms or software that are central to the research but not yet described in published literature, software must be made available to editors and reviewers. We strongly encourage code deposition in a community repository (e.g. GitHub). See the Nature Portfolio guidelines for submitting code & software for further information.

Data

Policy information about availability of data

All manuscripts must include a data availability statement. This statement should provide the following information, where applicable:

- Accession codes, unique identifiers, or web links for publicly available datasets
- A description of any restrictions on data availability
- For clinical datasets or third party data, please ensure that the statement adheres to our policy

Data analysis was performed with standard protocols of analysis tools. No custom code was used in this manuscript.
The RNA-seq data generated in this study have been deposited in the NCBI GEO database under accession code GSE167699 (https://www.ncbi.nlm.nih.gov/geo/query/acc.cgi?acc=GSE167699).
The mass spectrometry data generated in this study have been deposited in the Integrated Proteome Resources database under accession code PXD037718 (https://www.iprox.cn/page/project.html?id=IPX0005282000).
Mus Mouse database used for MS analysis was downloaded from Uniprot.
All plasmids and experimental materials in this study are available from the corresponding author upon request.
Source data are provided with this paper.

Human research participants

Policy information about studies involving human research participants and Sex and Gender in Research.

Reporting on sex and gender	N/A.
Population characteristics	N/A.
Recruitment	N/A.
Ethics oversight	N/A.

Note that full information on the approval of the study protocol must also be provided in the manuscript.

Field-specific reporting

Please select the one below that is the best fit for your research. If you are not sure, read the appropriate sections before making your selection.

☒ Life sciences ☐ Behavioural & social sciences ☐ Ecological, evolutionary & environmental sciences

For a reference copy of the document with all sections, see nature.com/documents/nr-reporting-summary-flat.pdf

Life sciences study design

All studies must disclose on these points even when the disclosure is negative.

Sample size	Sample size was similar to those used in this field: for the in vivo immunization, sample size was determined on the basis of prior knowledge of variability of similar experiments (PMID: 27481129, 33264617, 33380497, 27481093 etc); for in vitro experiments, a minimum of three samples were analyzed for most experiments, which were biologically repeated for at least three times in most experiments on the basis of prior knowledge of variability of similar experiments (PMID: 33264617, 27481093, 27261530, 27217539, 29026085 etc).
Data exclusions	No data was excluded in this study.
Replication	All experiments were done at least three times if necessary. The meaning of "n" in each figure is described in the figure legend in manuscript. All attempts for replication shows similar results.
Randomization	Randomization was applied wherever possible. For animal experiments, sex-matched and age-matched littermate mice in indicated genotypes were randomly assigned to different groups for immunization. For cellular experiments, cells of different genotype were parallel seeded and randomly assigned for further treatment. Besides, randomization was not performed due to samples needed to be grouped in specific orders for final figures.
Blinding	Researchers were blinded to the experimental groups of the iPCs differentiation from different genotypes naïve B cells, and also the in vivo immunization experiments. Sample collection and processing were labelled as code names and operated by different individual until assessing outcome. Otherwise, researchers were not blinded because it was necessary to allocate samples to experimental groups based on differences intrinsic to the samples.

Behavioural & social sciences study design

All studies must disclose on these points even when the disclosure is negative.

Study description	*Briefly describe the study type including whether data are quantitative, qualitative, or mixed-methods (e.g. qualitative cross-sectional, quantitative experimental, mixed-methods case study).*
Research sample	*State the research sample (e.g. Harvard university undergraduates, villagers in rural India) and provide relevant demographic information (e.g. age, sex) and indicate whether the sample is representative. Provide a rationale for the study sample chosen. For studies involving existing datasets, please describe the dataset and source.*
Sampling strategy	*Describe the sampling procedure (e.g. random, snowball, stratified, convenience). Describe the statistical methods that were used to predetermine sample size OR if no sample-size calculation was performed, describe how sample sizes were chosen and provide a rationale for why these sample sizes are sufficient. For qualitative data, please indicate whether data saturation was considered, and what criteria were used to decide that no further sampling was needed.*
Data collection	*Provide details about the data collection procedure, including the instruments or devices used to record the data (e.g. pen and paper, computer, eye tracker, video or audio equipment) whether anyone was present besides the participant(s) and the researcher, and whether the researcher was blind to experimental condition and/or the study hypothesis during data collection.*
Timing	*Indicate the start and stop dates of data collection. If there is a gap between collection periods, state the dates for each sample cohort.*
Data exclusions	*If no data were excluded from the analyses, state so OR if data were excluded, provide the exact number of exclusions and the rationale behind them, indicating whether exclusion criteria were pre-established.*
Non-participation	*State how many participants dropped out/declined participation and the reason(s) given OR provide response rate OR state that no participants dropped out/declined participation.*
Randomization	*If participants were not allocated into experimental groups, state so OR describe how participants were allocated to groups, and if allocation was not random, describe how covariates were controlled.*

Ecological, evolutionary & environmental sciences study design

All studies must disclose on these points even when the disclosure is negative.

Study description	*Briefly describe the study. For quantitative data include treatment factors and interactions, design structure (e.g. factorial, nested, hierarchical), nature and number of experimental units and replicates.*
Research sample	*Describe the research sample (e.g. a group of tagged Passer domesticus, all Stenocereus thurberi within Organ Pipe Cactus National Monument), and provide a rationale for the sample choice. When relevant, describe the organism taxa, source, sex, age range and any manipulations. State what population the sample is meant to represent when applicable. For studies involving existing datasets, describe the data and its source.*
Sampling strategy	*Note the sampling procedure. Describe the statistical methods that were used to predetermine sample size OR if no sample-size calculation was performed, describe how sample sizes were chosen and provide a rationale for why these sample sizes are sufficient.*
Data collection	*Describe the data collection procedure, including who recorded the data and how.*
Timing and spatial scale	*Indicate the start and stop dates of data collection, noting the frequency and periodicity of sampling and providing a rationale for these choices. If there is a gap between collection periods, state the dates for each sample cohort. Specify the spatial scale from which the data are taken*
Data exclusions	*If no data were excluded from the analyses, state so OR if data were excluded, describe the exclusions and the rationale behind them, indicating whether exclusion criteria were pre-established.*
Reproducibility	*Describe the measures taken to verify the reproducibility of experimental findings. For each experiment, note whether any attempts to repeat the experiment failed OR state that all attempts to repeat the experiment were successful.*
Randomization	*Describe how samples/organisms/participants were allocated into groups. If allocation was not random, describe how covariates were controlled. If this is not relevant to your study, explain why.*
Blinding	*Describe the extent of blinding used during data acquisition and analysis. If blinding was not possible, describe why OR explain why blinding was not relevant to your study.*

Did the study involve field work? ☐ Yes ☐ No

Field work, collection and transport

Field conditions	*Describe the study conditions for field work, providing relevant parameters (e.g. temperature, rainfall).*
Location	*State the location of the sampling or experiment, providing relevant parameters (e.g. latitude and longitude, elevation, water depth).*
Access & import/export	*Describe the efforts you have made to access habitats and to collect and import/export your samples in a responsible manner and in compliance with local, national and international laws, noting any permits that were obtained (give the name of the issuing authority, the date of issue, and any identifying information).*
Disturbance	*Describe any disturbance caused by the study and how it was minimized.*

Reporting for specific materials, systems and methods

We require information from authors about some types of materials, experimental systems and methods used in many studies. Here, indicate whether each material, system or method listed is relevant to your study. If you are not sure if a list item applies to your research, read the appropriate section before selecting a response.

Materials & experimental systems

n/a	Involved in the study	
☐	☒	Antibodies
☐	☒	Eukaryotic cell lines
☒	☐	Palaeontology and archaeology
☐	☒	Animals and other organisms
☒	☐	Clinical data
☒	☐	Dual use research of concern

Methods

n/a	Involved in the study	
☒	☐	ChIP-seq
☐	☒	Flow cytometry
☒	☐	MRI-based neuroimaging

Antibodies

Antibodies used

Biolegend: https://www.biolegend.com/
1.APC anti-mouse CD21, Cat#123412,1:200, Clone name: 7E9
2.APC anti-mouse CD117, Cat#105812, 1:200, Clone name: 2B8
3.APC anti-mouse GL7, Cat#144617, 1:200, Clone name: GL7
4.APC-Cyanine7 anti-mouse CD69, Cat#104525, 1:200, Clone name: H1.2F3
5.Biotin anti-CD5, Cat#100604, 1:200, Clone name: 53-7.3
6.Biotin anti-CD43, Cat#121204, 1:200, Clone name: 1B11
7.Biotin anti-Ter119, Cat#116204, 1:200, Clone name: TER-119
8.FITC anti-mouse CD43, Cat#143204, 1:200, Clone name: S11
9.eFluor 450 anti-mouse B220, Cat#103227, 1:200, Clone name: RA3-6B2
10.PE anti-mouse CD23, Cat#101608, 1:200, Clone name: B3B4
11.PE anti-mouse CD25 , Cat#102008, 1:200, Clone name: PC61

eBioscience: https://www.fishersci.com/us/en/brands/ITA5Q42L/ebioscience.html
1.APC anti-mouse CD19, Cat#17-0193-82, 1:200, Clone name: eBio1D3
2.APC-eFluor™ 780 anti-mouse IgM, Cat#47-5790-80), 1:200, Clone name: II/41
3.Anti-CD40, Cat#16-0402-86, 1:200, Clone name: HM40-3
4.Biotin anti-mouse CD93, Cat#13-5892-85, 1:200, Clone name: AA4.1
5.eFluor 450 anti-mouse CD19, Cat#48-0193-82, 1:200, Clone name: eBio1D3
6.PE anti-mouse CD5, Cat#12-0051-82, 1:200, Clone name: 53-7.3
7.PE anti-mouse CD86 , Cat#12-0862-83, 1:200, Clone name: GL1
8.PE-Cyanine7 anti-mouse CD3e, Cat#25-0031-82, 1:200, Clone name: 145-2C11
9.PE-Cyanine7 anti-mouse CD80, Cat#25-0801-82, 1:200, Clone name: 16-10A1
10.PerCP-CY5.5 anti-mouse B220, Cat#45-0452-82, 1:200, Clone name: RA3-6B2
11.Streptavidin APC-eFluor™ 780, Cat#47-4317-82, 1:200

BD Pharmingen™: https://us.vwr.com/
1.APC-CY7 anti-mouse CD45.1, Cat#560579, 1:200, Clone name: Clone name: A20

BD Biosciences: https://www.bdbiosciences.com/
1.Biotin anti-mouse CD9, Cat#558749, 1:200, Clone name: Clone name: KMC8
2.APC anti-mouse CD138, Cat#558626), 1:200, Clone name: Clone name: 281-2
3.Biotin anti-mouse CD138, Cat#553713, 1:100, Clone name: 281-2
4.FITC anti-mouse CD95, Cat#561979, 1:200, Clone name: Jo2

5.PE anti-mouse BLIMP1, Cat#564702,1:100, Clone name: 6D3
6.PerCP-CY5.5 anti-mouse CD45.2, Cat#552950, 1:200, Clone name: 104

Southern biotech :https://www.southernbiotech.com/
Mouse IgE-UNLB, Cat#0114-01, Gradient dilution (1:50-1:1000000), Clone name: 15.3
Mouse IgG1-UNLB, Cat#0102-01, Gradient dilution (1:50-1:1000000), Clone name: 15H6
Mouse IgG3-UNLB, Cat#0105-01, Gradient dilution (1:50-1:1000000), Clone name: B10
Mouse IgM-UNLB, Cat#0101-01), Gradient dilution (1:50-1:1000000), Clone name: 11E10
goat anti-mouse IgE-BIOT, Cat#1110-08,1:500
goat anti-mouse IgG1-BIOT, Cat#1070-08,1:500
goat anti-mouse IgG3-BIOT, Cat#1100-08,1:500
goat anti-mouse IgM-BIOT, Cat#1020-08,1:500
goat anti-mouse Kappa-UNLB, Cat#1050-01,1:200
goat anti-mouse Kappa-UNLB, Cat#1060-01),1:200

CST: https://www.cellsignal.com/
1.Anti-p38α, Cat#9228S, 1:1000, Clone name: L53F8
2.Anti-phospho-p38(Thy180/Tyr182) , Cat#9211S, 1:500 (polyclone)

Proteintech: https://www.ptgcn.com/
1.Anti-TCF3, Cat#21242-1-AP, 1:1000 (polyclone)
2.Anti-IRF4, Cat#11247-2-AP, 1:1000 (polyclone)
3.Anti-TCF4, Cat#22337-1-AP, 1:1000 (polyclone)

Santa Cruz: https://www.santacruzbicycles.com/
1.Anti-Actin, Cat#sc-47778, 1:1000, Clone name: C4
2.Anti-BLIMP1, Cat#sc-47732, 1:1000, Clone name: 6D3

ABclonal: https://abclonal.com/
1.Anti-GAPDH, Cat#AC002, 1:1000, Clone name: AC002

AbD Serotec: https://www.selectscience.net/
1.Anti-IgM, Cat#MCA1293,1:1000, Clone name: LO-MM-3

Validation

Antibodies used in this work were validated for its use by manufacturers. Primary antibodies (p38α, p-p38α, TCF3, TCF4, IRF4, BLIMP1) validation were based on data sheets provided by manufacturers and confirmed in this study by using overexpression and KO/KD Western-Blot samples.

Other commercially available antibodies were validated by the company, as well as other researchers (information collected by the RRID database):
APC anti-mouse CD21 (Cat#123412, RRID:AB_2085160), APC anti-mouse CD117 (Cat#105812, RRID:AB_313221), APC anti-mouse GL7 (Cat#144617, RRID:AB_2800674), APC-Cyanine7 anti-mouse CD69 (Cat#104525, RRID:AB_10683447), biotin anti-CD5 (Cat#100604, RRID:AB_312733), biotin anti-CD43 (Cat#121204, RRID:AB_493384), biotin anti-Ter119 (Cat#116204, RRID:AB_313705), FITC anti-mouse CD43 (Cat#143204, RRID:AB_10960745), eFluor 450 anti-mouse B220 (Cat#103227, RRID:AB_492876), PE anti-mouse CD23 (Cat#101608, RRID:AB_312833), PE anti-mouse CD25 (Cat#102008, RRID:AB_312857), APC anti-mouse CD19 (Cat#17-0193-82, RRID:AB_1659676), APC-eFluor™ 780 anti-mouse IgM (Cat#47-5790-80, RRID:AB_2573983), anti-CD40 (Cat#16-0402-86, RRID:AB_468947), biotin anti-mouse CD93 (Cat#13-5892-85, RRID:AB_466768), eFluor 450 anti-mouse CD19 (Cat#48-0193-82, RRID:AB_2734905), PE anti-mouse CD5 (Cat#12-0051-82, RRID:AB_465523), PE anti-mouse CD86 (Cat#12-0862-83, RRID:AB_465769), PE-Cyanine7 anti-mouse CD3e (Cat#25-0031-82, RRID:AB_469572), PE-Cyanine7 anti-mouse CD80 (Cat#25-0801-82, RRID:AB_2573370), PerCP-CY5.5 anti-mouse B220 (Cat#45-0452-82, RRID:AB_1107006),APC-CY7 anti-mouse CD45.1 (Cat#560579, RRID:AB_1727487), Biotin anti-mouse CD9 (Cat#558749, RRID:AB_397103), APC anti-mouse CD138 (Cat#558626, RRID:AB_1645216), Biotin anti-mouse CD138 (Cat#553713, 394999), FITC anti-mouse CD95 (Cat#561979, RRID:AB_10892808), PE anti-mouse BLIMP1 (Cat#564702, RRID:AB_2738901), PerCP-CY5.5 anti-mouse CD45.2 (Cat#552950, RRID:AB_394528), Mouse IgE-UNLB (Cat#0114-01, RRID:AB_2793972), IgG1-UNLB (Cat#0102-01, RRID:AB_2793845), IgG3-UNLB (Cat#0105-01, RRID:AB_2793898), IgM-UNLB (Cat#0101-01, RRID:AB_2629437), Anti-p38α (Cat#9228S, RRID:AB_490886), Anti-phospho-p38(Thy180/Tyr182) (Cat#9211S, RRID:AB_331641), Anti-TCF3 (Cat#21242-1-AP, RRID:AB_10755295), Anti-IRF4 (Cat#11247-2-AP, RRID:AB_2264940), Anti-TCF4 (Cat#22337-1-AP, RRID:AB_2879076), Anti-Actin (Cat#sc-47778, RRID:AB_626632), Anti-BLIMP1 (Cat#sc-47732, RRID:AB_628168), Anti-GAPDH (Cat#AC002, RRID:AB_2736879), Anti-IgM (Cat#MCA1293, RRID:AB_321868).

Eukaryotic cell lines

Policy information about cell lines and Sex and Gender in Research

Cell line source(s)

HEK293T and NIH3T3 cells were from American Type Culture Collection center (ATCC), and 293A(p38-/-) cells was a kindly gift from Prof. KL Guan at USCD (PMID: 28752853).

Authentication

HEK293T cells and NIH3T3 cells were pre-authenticated by ATCC by STR sequencing. 293A(p38-/-) cells were confirmed by Western blot.

Mycoplasma contamination

No cell lines were positive for Mycoplasma contamination in a PCR test method.

Commonly misidentified lines<br>(See ICLAC register)	No commonly misidentified lines were used.

Palaeontology and Archaeology

Specimen provenance	*Provide provenance information for specimens and describe permits that were obtained for the work (including the name of the issuing authority, the date of issue, and any identifying information). Permits should encompass collection and, where applicable, export.*
Specimen deposition	*Indicate where the specimens have been deposited to permit free access by other researchers.*
Dating methods	*If new dates are provided, describe how they were obtained (e.g. collection, storage, sample pretreatment and measurement), where they were obtained (i.e. lab name), the calibration program and the protocol for quality assurance OR state that no new dates are provided.*

☐ Tick this box to confirm that the raw and calibrated dates are available in the paper or in Supplementary Information.

Ethics oversight	*Identify the organization(s) that approved or provided guidance on the study protocol, OR state that no ethical approval or guidance was required and explain why not.*

Note that full information on the approval of the study protocol must also be provided in the manuscript.

Animals and other research organisms

Policy information about studies involving animals; ARRIVE guidelines recommended for reporting animal research, and Sex and Gender in Research

Laboratory animals	p38αfl/fl (Jax stock, 031129) was previously described. p38β-/-, p38γ-/-, p38δ-/- mice were a kindly gift from Huiping Jiang (Boehringer Ingelheim Pharmaceuticals, Inc, Ridgefield, Connecticut). Vavcre (Jax stock, 008610), CD19cre (Jax stock, 006785), C57BL/6J (Jax stock, 000664), Tcf3fl/fl (isolated from Jax stock, 024511), Irf4fl/fl (Jax stock, 009380), Blimp1fl/fl (Jax stock, 008100), CD45.1 B6.SJL (Jax stock, 002014), Cas9tg/+ (Jax stock, 026179) mice were purchased from the Jackson Laboratory. Blimp1gfp/+ mice were a kind gift from Stephen L. Nutt. All the mice were back-crossed to the C57BL/6J background for at least 6 generations, and housed in a specific pathogen-free facility under a 12h light-dark cycle at the Xiamen University Laboratory Animal Center.The light time was from 8 a.m. to 8 p.m., and the room temperature were kept at 22-24 °C and humidity at 50-70%. Unless stated otherwise (5-6 weeks old mice used for analysis B cells development), both male and female mice (8-10 weeks old) were used in this study.
Wild animals	This study did not involve wild animals.
Reporting on sex	Mice of a single sex were used only when sufficient mice were available. Otherwise, mice of both sexes were used. The findings apply to mice of both sexes.
Field-collected samples	This study did not involve samples collected from the field.
Ethics oversight	All the mice were housed in a specific pathogen-free facility under a 12h light-dark cycle at the Xiamen University Laboratory Animal Center. All mouse experiments were approved by the Animal Care and Use Committee of Xiamen University (XMULAC20180073).

Note that full information on the approval of the study protocol must also be provided in the manuscript.

Clinical data

Policy information about clinical studies

All manuscripts should comply with the ICMJE guidelines for publication of clinical research and a completed CONSORT checklist must be included with all submissions.

Clinical trial registration	*Provide the trial registration number from ClinicalTrials.gov or an equivalent agency.*
Study protocol	*Note where the full trial protocol can be accessed OR if not available, explain why.*
Data collection	*Describe the settings and locales of data collection, noting the time periods of recruitment and data collection.*
Outcomes	*Describe how you pre-defined primary and secondary outcome measures and how you assessed these measures.*

Dual use research of concern

Policy information about dual use research of concern

Hazards

Could the accidental, deliberate or reckless misuse of agents or technologies generated in the work, or the application of information presented in the manuscript, pose a threat to:

No	Yes	
☐	☐	Public health
☐	☐	National security
☐	☐	Crops and/or livestock
☐	☐	Ecosystems
☐	☐	Any other significant area

Experiments of concern

Does the work involve any of these experiments of concern:

No	Yes	
☐	☐	Demonstrate how to render a vaccine ineffective
☐	☐	Confer resistance to therapeutically useful antibiotics or antiviral agents
☐	☐	Enhance the virulence of a pathogen or render a nonpathogen virulent
☐	☐	Increase transmissibility of a pathogen
☐	☐	Alter the host range of a pathogen
☐	☐	Enable evasion of diagnostic/detection modalities
☐	☐	Enable the weaponization of a biological agent or toxin
☐	☐	Any other potentially harmful combination of experiments and agents

ChIP-seq

Data deposition

☐ Confirm that both raw and final processed data have been deposited in a public database such as GEO.

☐ Confirm that you have deposited or provided access to graph files (e.g. BED files) for the called peaks.

Data access links<br>*May remain private before publication.*	*For "Initial submission" or "Revised version" documents, provide reviewer access links. For your "Final submission" document, provide a link to the deposited data.*
Files in database submission	*Provide a list of all files available in the database submission.*
Genome browser session<br>(e.g. UCSC)	*Provide a link to an anonymized genome browser session for "Initial submission" and "Revised version" documents only, to enable peer review. Write "no longer applicable" for "Final submission" documents.*

Methodology

Replicates	*Describe the experimental replicates, specifying number, type and replicate agreement.*
Sequencing depth	*Describe the sequencing depth for each experiment, providing the total number of reads, uniquely mapped reads, length of reads and whether they were paired- or single-end.*
Antibodies	*Describe the antibodies used for the ChIP-seq experiments; as applicable, provide supplier name, catalog number, clone name, and lot number.*
Peak calling parameters	*Specify the command line program and parameters used for read mapping and peak calling, including the ChIP, control and index files used.*
Data quality	*Describe the methods used to ensure data quality in full detail, including how many peaks are at FDR 5% and above 5-fold enrichment.*
Software	*Describe the software used to collect and analyze the ChIP-seq data. For custom code that has been deposited into a community repository, provide accession details.*

Flow Cytometry

Plots

Confirm that:

☒ The axis labels state the marker and fluorochrome used (e.g. CD4-FITC).

☒ The axis scales are clearly visible. Include numbers along axes only for bottom left plot of group (a 'group' is an analysis of identical markers).

☒ All plots are contour plots with outliers or pseudocolor plots.

☒ A numerical value for number of cells or percentage (with statistics) is provided.

Methodology

Sample preparation	Cells from spleen was grinded gently and single-cell suspension was collected by filtering with 70 µM filters and RBCs were depleted with ammonium chloride lysis buffer (Beyotime, C3702). In vitro cultured cells were also collected by filtering with 70 µM filters. For cell surface marker staining, cells were incubated with antibodies in 1x FACS buffer (1% FBS in PBS) for 30 mins at 4 °C, and washed with 1x FACS buffer twice before analysis. For intracellular staining, surface marker-stained cells were fixed with Fixation/Permeabilization buffer (BD, 554714) for 10 mins at room temperature, washed with 1x Permeabilization buffer, and treated with indicated antibodies following the manufacturer's instructions. All the stained samples were kept at 4 °C for indicated analysis.
Instrument	Acquisition: BD fortesa X20;<br>BD Aril-FUSION.
Software	FlowJo (version: 10.6.2).
Cell population abundance	Cell abundance were labelled in related figures in the manuscript, and majors were listed below:<br>iGCBs: >95% in the iPC culture at day 4; 3%-50%<br>iPCs: 10%-60% in the iPC culture at day 8, including KO cells;<br>iPCs: 3%-50% after LPS stimulation at day 4, including KO cells;<br>GCB cells in OVA/Alum/LPS-immunized mice: 7%-12% in splenocyte.<br>PCs in OVA/Alum/LPS-immunized mice: 0.5%-5% in splenocyte.<br>NP-specific GCB cells in NP-OVA/Alum-immunized mice: 0.07%-0.15% in splenocyte.<br>NP-specific PBs in NP-OVA/Alum-immunized mice: 0.001%-0.01% in splenocyte.<br>NP-specific PBs in NP-Ficoll-immunized mice: 0.01%-0.03% in splenocyte.<br>NP-specific PBs in NP-LPS-immunized mice: 0.002%-0.04% in splenocyte.<br>Mature B in bone marrow of non-immunized mice: 0.5%-4%.
Gating strategy	Events were gated by size on FSC(H) /FSC(A) and SSC(H)/ SSC(A) to exclude cell debris, then double exclusion on FSC-W/H and SSC-W/H and exclusion of dead cells. Different subgroup cells were gated base on the indicated dyes described in the manuscript.

☒ Tick this box to confirm that a figure exemplifying the gating strategy is provided in the Supplementary Information.

Magnetic resonance imaging

Experimental design

Design type	*Indicate task or resting state; event-related or block design.*
Design specifications	*Specify the number of blocks, trials or experimental units per session and/or subject, and specify the length of each trial or block (if trials are blocked) and interval between trials.*
Behavioral performance measures	*State number and/or type of variables recorded (e.g. correct button press, response time) and what statistics were used to establish that the subjects were performing the task as expected (e.g. mean, range, and/or standard deviation across subjects).*

Acquisition

Imaging type(s)	*Specify: functional, structural, diffusion, perfusion.*
Field strength	*Specify in Tesla*
Sequence & imaging parameters	*Specify the pulse sequence type (gradient echo, spin echo, etc.), imaging type (EPI, spiral, etc.), field of view, matrix size, slice thickness, orientation and TE/TR/flip angle.*
Area of acquisition	*State whether a whole brain scan was used OR define the area of acquisition, describing how the region was determined.*

Diffusion MRI ☐ Used ☐ Not used

Preprocessing

Preprocessing software	*Provide detail on software version and revision number and on specific parameters (model/functions, brain extraction, segmentation, smoothing kernel size, etc.).*
Normalization	*If data were normalized/standardized, describe the approach(es): specify linear or non-linear and define image types used for transformation OR indicate that data were not normalized and explain rationale for lack of normalization.*
Normalization template	*Describe the template used for normalization/transformation, specifying subject space or group standardized space (e.g. original Talairach, MNI305, ICBM152) OR indicate that the data were not normalized.*
Noise and artifact removal	*Describe your procedure(s) for artifact and structured noise removal, specifying motion parameters, tissue signals and physiological signals (heart rate, respiration).*
Volume censoring	*Define your software and/or method and criteria for volume censoring, and state the extent of such censoring.*

Statistical modeling & inference

Model type and settings	*Specify type (mass univariate, multivariate, RSA, predictive, etc.) and describe essential details of the model at the first and second levels (e.g. fixed, random or mixed effects; drift or auto-correlation).*
Effect(s) tested	*Define precise effect in terms of the task or stimulus conditions instead of psychological concepts and indicate whether ANOVA or factorial designs were used.*

Specify type of analysis: ☐ Whole brain ☐ ROI-based ☐ Both

Statistic type for inference (See Eklund et al. 2016)	*Specify voxel-wise or cluster-wise and report all relevant parameters for cluster-wise methods.*
Correction	*Describe the type of correction and how it is obtained for multiple comparisons (e.g. FWE, FDR, permutation or Monte Carlo).*

Models & analysis

n/a	Involved in the study
☐	☐ Functional and/or effective connectivity
☐	☐ Graph analysis
☐	☐ Multivariate modeling or predictive analysis

Functional and/or effective connectivity	*Report the measures of dependence used and the model details (e.g. Pearson correlation, partial correlation, mutual information).*
Graph analysis	*Report the dependent variable and connectivity measure, specifying weighted graph or binarized graph, subject- or group-level, and the global and/or node summaries used (e.g. clustering coefficient, efficiency, etc.).*
Multivariate modeling and predictive analysis	*Specify independent variables, features extraction and dimension reduction, model, training and evaluation metrics.*
